# The Effect of Early Neighborhood Contexts on Cognitive Function in Midlife

**DOI:** 10.1093/geroni/igab046.1515

**Published:** 2021-12-17

**Authors:** Heewon Yoon, Jean Choi, Giancarlo Pasquini, Alexa Allan, Martin Sliwinski, Stacey Scott, Elizabeth Munoz

**Affiliations:** 1 University of Texas at Austin, Austin, Texas, United States; 2 The University of Texas at Austin, Austin, Texas, United States; 3 Stony Brook University, Stony Brook, New York, United States; 4 Pennsylvania State University, University Park, Pennsylvania, United States; 5 The Pennsylvania State University, University Park, Pennsylvania, United States; 6 The University of Texas at Austin, The University of Texas at Austin, Texas, United States

## Abstract

We evaluated associations between objective and subjective early-life neighborhood contexts and cognitive function at midlife. Study participants grew up in different addresses but resided in the same urban zip code at the time of data collection thus controlling for concurrent neighborhood contexts. Participants provided their home address when they were five-years-old and recalled their age-five neighborhood conditions (Mage= 40.59 (7.91); n = 130). Age-five addresses were geocoded and linked with harmonized longitudinal Census tract boundaries and variables. Predictive models with a self-reported neighborhood conditions score, an objective neighborhood deprivation indicator, and other sociodemographic covariates indicated that poorer age-five self-reported neighborhood conditions were significantly associated with lower baseline (Cohen’s d = -.24) and average daily (d = -.21) working memory performance. There were no associations with objective age-five neighborhoods. Results contribute to a growing literature on the role of psychosocial neighborhood contexts on cognition that may extend back to childhood neighborhoods.

